# Analgesia-free flexible ureteroscopic treatment and laser lithotripsy for removal of a large urinary stone: a case report

**DOI:** 10.1186/s13256-015-0699-0

**Published:** 2015-10-02

**Authors:** Konrad Wilhelm, Alexander Frankenschmidt, Arkadiusz Miernik

**Affiliations:** Department of Urology, University Medical Center, Hugstetterstr. 55, D-79106 Freiburg, Germany

**Keywords:** Continent urinary diversion, Endourology, Laser lithotripsy, Ureteral access sheath, Urolithiasis

## Abstract

**Introduction:**

Urinary stone formation is a frequent complication after continent urinary tract diversion and can require complex surgical management. Therapy options include open, percutaneous, transurethral, or transstomal stone fragmentation and extraction. The transstomal approach is considered to be one of the more complex treatment modalities. The patient’s individual anatomy, minor stoma diameter, and the existing continence mechanism in the majority of cases cause substantial technical challenges for the surgeon. We present here what we believe to be the first description of an analgesia-free flexible endoscopic removal of a large pouch stone in an out-patient care setting. Additionally, we provide a brief overview of competing techniques.

**Case presentation:**

A 30-year-old Caucasian woman with a history of lower urinary tract reconstruction with an ileal pouch and a continent umbilical stoma was admitted to our department with pouch urolithiasis in the urinary reservoir. We employed a minimally invasive approach to extract the stone using flexible ureteroscopy via a modified access sheath and laser lithotripsy. No analgesia is needed with this procedure and it can be performed in an out-patient setting.

**Conclusion:**

The described clinical case highlights the difficulties of treating this high-incidence problem in patients with continent urinary diversions. Our presented technique is of particular interest to urologists and family doctors, and could improve the treatment of such patients by lowering the morbidity of the intervention.

## Introduction

Urolithiasis is a frequent clinical problem after continent urinary diversion [1]. Shock wave lithotripsy (SWL), open, and endoscopic techniques have been found to be successful in the management of calculi within different urinary reservoirs [2, 3]. The majority of authors discourage clinicians from using transstomal approaches because of possible damage to the continence mechanism [1, 4]. The percutaneous or open approaches can also be challenging and carry a high risk of complications because most patients with a urinary diversion have a history of multiple previous abdominal surgeries. Furthermore, these options need general anesthesia, hospitalization, and often prolonged postoperative catheterization. However, miniaturization of the endoscopic armamentarium might revive the concept of transstomal therapy as a safe and efficient option for stone extraction, and even provide an analgesia-free therapy option.

## Case presentation

**Fig. 1:**
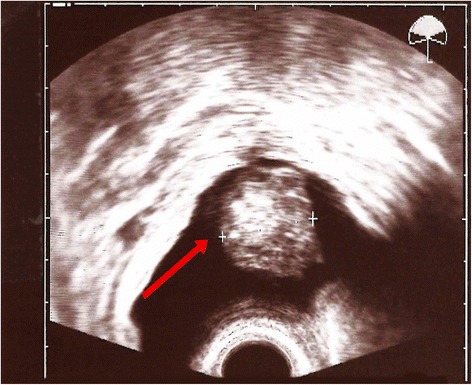
Transvaginal ultrasound of the continent urinary reservoir showing a 3cm calculus. The arrow points to the big calculus in the continent urinary reservoir.

Our patient was a 30-year-old Caucasian woman with history of bladder extrophy in childhood followed by multiple reconstructive surgeries, including ileal urinary diversion. Urine continence was maintained by a detubularized ileal segment according to the technique of Monti *et al*. [5]. After her initial surgical extrophy treatment, including urinary diversion, more than 20 additional abdominal interventions were needed because of stoma incontinence, prolapse, adhesions, and reservoir fistula. The last procedure was performed in 2012 owing to via falsa formation within the Monti-segment (MS). Since that time, continence had remained satisfactory and our patient could empty urine amounts of 500–700ml by clean intermittent catheterization with a 14F catheter four to five times a day. Besides occasional urine loss, our patient reported no other abnormal conditions. During a routine transvaginal ultrasonography in September 2014, a suspicious echogenic calculus formation was revealed at the bottom of the urinary reservoir (Fig. 1) and she was therefore admitted to our urology department. Flexible endoscopic inspection using a conventional 9.8F ureteroscope was conducted via the MS, confirming the presence of a single 3cm stone.

Considering the different treatment options and the individual circumstances, we decided to carry out a flexible endoscopy in an additional session.

**Fig. 2:**
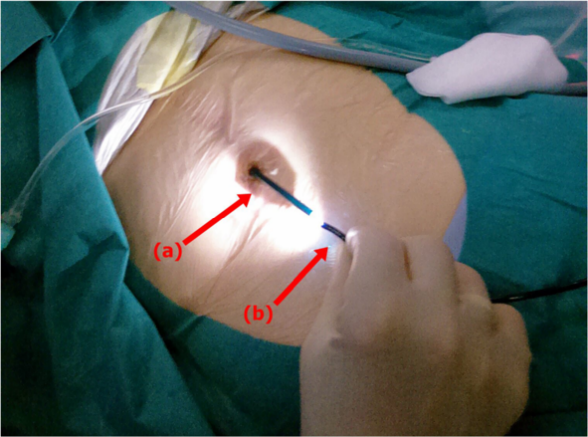
Intraoperative view demonstrating the instruments used for transstomal stone lithotripsy showing a modified ureteral access sheath (*a*) and a conventional flexible ureteroscope (*b*)

**Fig. 3:**
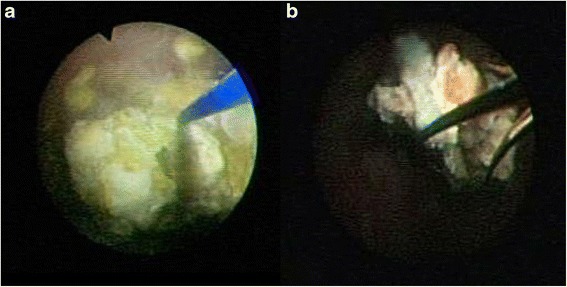
Endoscopic view describing treatment progress. **a** Sphinx YAG:holmium laser stone fragmentation. **b** Removal of the fragments using an open-tip extractor

The treatment was performed with our patient in the supine position without general or local anesthetics. Her paraumbilical region was disinfected and covered with sterile surgical draping. The MS was lubricated and a hydrophilic guide wire inserted. For stoma protection against extensive endoscopic manipulation, a standard 12/14F ureteral access sheath (UAS) was shortened to 15cm length and gently placed over the wire. After insertion of the flexible ureteroscope, calculus disintegration was performed using a holmium laser system with thin fibers (274μm) in dusting mode (0.6J, 15Hz, short pulse length). Larger fragments were removed using an NGage open-tip extractor (Cook Medical, Bloomington, IN, USA). The remaining material was washed out using a 10F catheter attached to a 50ml syringe. Potential laser-related lesions and/or rest fragments were excluded by endoscopy (Figs. 2 and 3). After removal of the UAS, a standard latex 14F urethral catheter was inserted using a guide wire, and left *in situ* for 4 days.

Our patient did not experience any significant pain during the intervention and was discharged on the same day. She reported full continence and no catheterization problems directly after the procedure and at follow-up 3 months later. Abdominal sonography showed no residual fragments. Infrared-based compositional analysis revealed calcium-carbonate-phosphate stone material.

Owing to high stone recurrence rates of up to 65% within 5 years in patients after continent urinary diversion, we recommended metaphylactic measures (increased daily fluid intake, short catheterization intervals, and self-monitoring for bacterial colonization) for our patient [6].

## Discussion

Stone formation in urinary reservoirs is a frequent problem, with reported incidences of 16–33% [1]. Contributing factors are urinary stasis, increased mucus production, bacterial colonization or infection, foreign bodies, suture material, poor compliance of patients with clean intermittent catheterization techniques, and metabolic disturbances if intestinal segments are involved.

Different treatment modalities have been described. Open surgery is possible but requires extended catheterization times and hospitalization. The increased risk of complications impedes the use of this method for patients after multiple abdominal surgeries [2]. SWL is feasible but often requires additional endoscopic fragment removal [7]. Percutaneous approaches utilizing Amplatz sheaths, UASs, and laparoscopic trocars have been described as excellent options [2, 8]. However, establishing a percutaneous tract by puncturing and dilating the abdominal wall and urinary reservoir seems to be considerably invasive and challenging, especially after multiple surgeries. The transstomal approach has been discussed critically in the literature with regard to the risk of stricture or permanent impairment of continence, This technique requires numerous stoma passages and excessive manipulation within the urinary diversion [4, 9].

Ongoing miniaturization of the endoscopic armamentarium and the introduction of new flexible devices might change the clinical management of such cases. UASs have been developed for retrograde intrarenal surgery to avoid multiple, potentially traumatic ureter passages. UASs protect the ureter wall and facilitate intrarenal manipulation, increasing irrigation volume and decreasing intrapelvic pressure [10]. Using a UAS to protect the reservoir stoma and the continence mechanism during an endoscopic intervention has been described recently in a pediatric case [11]. A calculus of unknown size was treated by laser fragmentation over a rigid 8.5F cystoscope. The UAS was shortened accordingly to suit to the length of the scope. The patient in that case was under general anesthesia.

To further reduce the invasiveness, we modified this approach using a flexible scope and a special open-tip extraction basket. Owing to the large stone mass, we excluded SWL as a treatment option. Open surgery and percutaneous access also seemed to be risky after the multiple abdominal surgeries in our patient. To the best of our knowledge, this is the first description of an adult patient with a large stone in the continent urinary reservoir after lower urinary tract reconstruction that was treated with a transstomal approach using a flexible ureteroscope and a modified UAS without anesthesia. The described technique enabled safe stone disintegration and removal of the fragments with maximum protection of the stoma and its continence mechanism. Compared to open surgery or percutaneous intervention, this technique is less invasive and avoids anesthesia and hospitalization. We did not encounter any of the problems discussed in the literature regarding transstomal manipulations, such as stricture or permanent negative impairment of continence. We suggest this safe and well-tolerated treatment modality should be considered in the clinical management of similar cases.

## Conclusion

Analgesia-free transstomal treatment of large stone fragments inside continent urinary reservoirs using a flexible ureteroscope and a modified UAS is feasible. Compared to open surgery or percutaneous intervention, it is a less invasive option and avoids anesthesia and hospitalization. This safe and well-tolerated treatment modality might be considered in the clinical management of urolithiasis in urinary reservoirs.

## Consent

Written informed consent was obtained from the patient for publication of this case report and accompanying images. A copy of the written consent is available for review by the Editor-in-Chief of this journal.

## References

[1] Beiko DT, Razvi H. Stones in urinary diversions: update on medical and surgical issues. Curr Opin Urol. 2002;12:297–303. 10.1097/00042307-200207000-00007 12072650

[2] Al-Marhoon MS, Sarhan OM, Awad BA, Helmy T, Ghali A, Dawaba MS. Comparison of endourological and open cystolithotomy in the management of bladder stones in children. J Urol. 2009;181:2684–8. 10.1016/j.juro.2009.02.040 19375100

[3] Cohen TD, Streem SB. Minimally invasive endourologic management of calculi in continent urinary reservoirs. Urology. 1994;43:865–8. 10.1016/0090-4295(94)90154-6 8197652

[4] James OL, Sung J, Marguet C, L’esperance A, Albala DM. The surgical management of stones in patients with urinary diversions. Curr Opin Urol. 2004;14:129–34. 10.1097/00042307-200403000-00014 15075843

[5] Monti PR, de Carvalho JR, Arap S. The Monti procedure: applications and complications. Urology. 2000;55:616–21. 10.1016/s0090-4295(99)00587-7 10792064

[6] Cohen TD, Streem SB, Lammert G. Long-term incidence and risks for recurrent stones following contemporary management of upper tract calculi in patients with a urinary diversion. J Urol. 1996;155:62–5. 7490899

[7] Claus C, Jorion JI, Libon E. Continent urinary diversion and calculus. Acta Urol Belg. 1997;65:33–7. 9497595

[8] Rhee AC, Cain MP. Percutaneous cystolithotomy in the pediatric neuropathic bladder with laparoscopic trocar access: a modified approach useful for the augmented and native bladder, and continent urinary reservoir. J Pediatr Urol. 2013;9:289–92. 10.1016/j.jpurol.2012.05.004 22795187

[9] DeFoor W, Minevich E, Reddy P, Sekhon D, Polsky E, Wacksman J, et al. Bladder calculi after augmentation cystoplasty: risk factors and prevention strategies. J Urol. 2004;172(5 Pt 1):1964–6. 10.1097/01.ju.0000140911.43898.15 15540766

[10] Miernik A, Schoenthaler M, Wilhelm K, Wetterauer U, Zyczkowski M, Paradysz A, et al. Combined semirigid and flexible ureterorenoscopy via a large ureteral access sheath for kidney stones >2cm: a bicentric prospective assessment. World J Urol. 2014;32:697–702. 10.1007/s00345-013-1126-z 23821122

[11] Vasudevan V, Strine AC, Kaefer M. A novel technique for endoscopic management of stones in a continent urinary reservoir. Urology. 2014;83:1398–400. 10.1016/j.urology.2014.02.012 24726150

